# TEM in the treatment of recurrent rectal cancer in the elderly

**DOI:** 10.1186/1471-2318-11-S1-A5

**Published:** 2011-08-24

**Authors:** G Benassai, S Perrotta, V Desiato, GL Benassai, O Mazzei, G Quarto

**Affiliations:** 1 Dipartimento di Chirurgia Generale, Geriatrica, Oncologica e Tecnologie Avanzate, Italy; 2 Facoltà di Medicina e Chirurgia, Università degli Studi di Napoli Federico II, Naples, Italy

## Introduction

Transanal endoscopic microsurgery is a useful technique of minimally invasive surgery that allows the realization of complex interventions, from transanal excisions to full thickness resections with anastomotic reconstructions.

TEM can have a diagnostic and therapeutic value in the elderly for the treatment of primary rectal cancer as well as for recurrences.

## Materials and methods

During the period between January 2002 and December 2009 six patients, average age of 66 years, four men and two women, with early diagnosed rectal cancer recurrence were selected to undergo this palliative surgical procedure. 3 men and 1 woman underwent "ultra-low anterior resection”, followed by chemo / radio therapy (T3N1M0); in one woman a TEM (T1NxM0) and in one old man the local escission was performed after neoadiuvant chemo/radio therapy (T2NxM0). The selection of the patients was made by: rigid sigmoidoscope, transrectal us, colonoscopy, abdominal us to rule out liver metastases, CT and MRI abdomen and pelvis with and without contrast agents, PET CT. In all patients the lesions were superficial, smaller than 2 cm and located at the posterior wall of the rectum.

## Results

Follow-up was approximately 12-28 months; the pathologic staging confirmed the complete excision of recurrences. Patients were then referred for more complementary therapies. Only one patient presented a retro rectal abscess treated with conservative techniques, too.

## Conclusions

The alternative to conservative surgery is an abdomino-perineal resection sec. Miles, but also this really invasive procedure can be considered palliative in the most part of recurrences. So, based on equal oncological results, the reduction of surgical trauma and preservation of anatomical integrity are really important goals.
